# Ultra-directional and high-efficiency µLEDs via gradient index filled micro-horn collimators

**DOI:** 10.1038/s41598-026-39920-7

**Published:** 2026-02-19

**Authors:** Alexander Luce, Rasoul Alaee, Aimi Abass

**Affiliations:** 1 Friedrich-Alexander-Universität Erlangen-Nürnberg, Erlangen, Germany; 2 ams-OSRAM, Regensburg, Germany

**Keywords:** µLED, nanoLED, FDTD, Near-eye AR/VR, Directionality, µDisplay, Light extraction efficiency, Outcoupling structure, Engineering, Optics and photonics, Physics

## Abstract

Micro-LEDs (µLEDs) are poised to transform near-eye AR/VR, display, and optical communication technologies, but they are currently hindered by low light extraction efficiency and non-directional emission. Our study introduces an innovative approach using a descending index multilayer anti-reflection coating combined with a horn collimator structure atop the µLED pixel. This design leverages the propagation of light outside the critical angle to enhance both the directionality and extraction efficiency of emitted light. By implementing either discrete or continuous refractive index gradients within the horn, we achieve a tenfold increase in light extraction within a $\pm 15^\circ$ cone, with an overall light extraction efficiency reaching approximately 80%, where 31% of the power is concentrated within this narrow cone. The enhancement is furthermore maintained across a broad range of Quantum well position variations. This performance surpasses that of an optimized SiO2 half-ellipsoidal lens, which diameter and height is 24X and 26X larger than the pixel width respectively, while our design only slightly increases the device height and expands the final light escape surface to 3 times and roughly 4 times the pixel width respectively. Such efficiency, directionality enhancement, and compactness make this solution particularly suitable for high-resolution, densely packed µLED arrays, promising advancements in high-performance, miniaturized display systems.

## Introduction

Generating light with high efficiency and directionality directly from the light source itself is in general a highly desirable characteristic for LEDs and for µLEDs^1–3^, in particular for near-eye AR/VR and display applications^4,5^ or optical communications^6,7^. µLEDs in particular are considered to be a promising so called light-engine to achieve high-efficiency, high-brightness and compact high-pixel-density displays which are required for near-eye AR/VR applications^8,9^. However, what technology will eventually emerge as the dominant light-engine for near-eye, full-colour AR/VR is still unclear^10,11^ and researchers are investigating micro-pixelated LCD displays^12^, OLEDs^5,13^, quantum dot conversion µLEDs^14^ or RBG-µLEDs^15^.

However, in addition to the difficulties in manufacturing and package integration of µLEDs^16–18^ current µLEDs still offer significant room for improvement in terms of efficiency, as suggested by the external quantum efficiency (EQE) of µLEDs which is typically still only in the range of a few percent^19^. Light, which is not outcoupled and remains trapped in the emitter, leads to secondary problems such as high power consumption, self-heating^20^ or pixel cross-talk^21^.

The EQE measures how much of the electrical energy injected into the LED is converted to useful light outside of the LED and is a crucial area of improvement for µLEDs^8,11,22^. It consists of three contributions, the electrical internal quantum efficiency ($\hbox {IQE}_e$), the Purcell factor (pf) and the total light extraction efficiency ($\eta _{\text {LEE}}$ = $\hbox {LEE}_{{90}}$). Often, purcell factor and electrical internal quantum efficiency is combined as $\eta _\text {IQE}$^23^. For many applications, only light at a particular solid angle $\Gamma$ is of interest, such as coupling into a fibre or waveguide which has a limited acceptance angle^24–26^ which is measured by $\hbox {LEE}_\Gamma$ whereas the fraction of light emitted into the solid angle $\Gamma$ over the total solid angle is denoted by $\eta _\Gamma$. For example, high directionality of the µLED is a requirement for a large field of view in diffractive waveguide couplers^9^ with solid angle of around $\Gamma =\pm 15^\circ$ being a typical target^27–29^.

As a consequence, all of these factors determine the overall performance of the µLED, the so called wall-plug-efficiency ($\eta _\text {WPE}$)^23^, and need to be maximized to achieve the overall best device performance. In general, the total wall-plug-efficiency of a µLED is therefore a product of different contributions

$$\begin{aligned} \eta _\text {WPE} = \eta _\text {EL}\,\eta _\text {IQE}\,\eta _\text {LEE}\,\eta _\Gamma , \end{aligned}$$

where $\eta _\text {EL}$ denotes the efficiency of the electrical system. The IQE of µLEDs remains a big challenge due to defect recombination at the interface of the active region with its surroundings^30,31^. In particular for the smallest µLEDs, the surface-to-bulk ratio becomes increasingly skewed and therefore a strain on the IQE due to extrinsic and intrinsic surface defects^32^.

To enhance the general light extraction efficiency of a LED or µLED, typical approaches involve utilizing resonant/lossy cavities with distributed-bragg-reflector- (DBR)^33^, anti-reflection-coatings^34^ and Gradient-index (GRIN) coatings^35,36^ or by introducing surface textures^19^. In particular anti-reflection-coatings can improve the direct outcoupling efficiency by reducing reflection losses at the interface. However, it is not possible to extract light with incidence angle inside the high refractive-index emitter beyond the total-internal-reflection (TIR) angle. DBRs, designed as incidence angle filters, can suppress emission at shallow angles but typically show higher losses due to absorption since the light interacts more often with absorbing materials inside the µLED pixel^23,37^.

Introducing textures on the exit surface of the µLED naturally breaks total internal reflection conditions and introduces scattering which in turn lead to increased light extraction^23^. While surface roughening increases the overall light extraction, it also leads to a broad, lambertian like far-field emission. However, for applications where only the light into a particular solid angle is useful, the lambertian-like farfield distributions results in low directionality which decreases the overall system efficiency for applications with directionality requirements^9,23^.

Light engines with highly directional, low-divergence emissions are generally preferred, as broad emission profiles significantly reduce coupling efficiency^22^. Because µLEDs naturally emit over a wide angle, considerable effort has focused on improving their emission directionality. Typical approaches involve micro-lens arrays^27,38,39^, surface texturing approaches^40^, meta-lenses^41–43^, µLED nanorods^44,45^ or photonic crystals^46–48^. In particular for the smallest µLEDs which are also the most interesting for near-eye AR/VR applications, increasing directionality and forward efficiency is especially difficult due to size of the devices. Traditional optical elements for collimation, such as lenses, behave differently for large LEDs due to the near-field, wave-optical properties of the µLED emission. This necessitates larger devices overall, which is in conflict with the compactness requirements of near-eye AR/VR applications^11^.

Photonic crystals and metasurfaces, particularly in the vicinity of the active region, can enhance emission directionality and even the spontaneous emission rate by allowing direct near-field coupling of dipole emitters to an engineered localized mode supported by the photonic crystal, which have the desired emission properties^29,48,49^. To ensure that the photonic crystal sustains the desired mode with favourable properties, one would need sufficiently many crystal lattice periods, which was recently shown to be on the order of $\sim$8 periods^48^. The directionality and outcoupling enhancement through such approaches are inherently position dependent and would require a minimal number of quantum wells, which in turn may limit the total performance as the enhancement may not be homogeneous across the whole active region (MQWs).

For the smallest monolithic pixels, die shaping, i.e. the process of altering the geometry of the pixel itself to shape the light appropriately^28,50^, has shown promising results for enhancing directionality and outcoupling efficiency. In particular, the sidewalls of the pixel geometry play a crucial role for efficiency and directionality of the µLED^30,51–54^.

Meanwhile, the problem of directionality has been thoroughly investigated in the field of microwave radiation and antenna technology where antenna geometries exists with large directionality and high efficiency^55^. However, there are significant differences between typical microwave antennas and µLEDs:

1. The wavelength of the radiation in µLEDs is small compared to the size of the device while the geometry is typically similar in size of the wavelength for microwaves applications.
2. The emission for µLEDs originate from an active region which is large compared to the wavelength.
3. The emission coming from different parts of the active region is incoherent due to the spontaneous emission process of radiative recombination^23^.
4. µLEDs exhibit a much greater number of available and populated modes due to the distributed nature of the emission from the active region and the overall size of the device compared to the wavelength.
5. In microwave applications, metals can be considered to be perfect electric conductors while they have considerable penetration depth and absorption at optical wavelengths for µLEDs.

In this work, we investigate the application of a so called µHorn collimator/antenna^55^ in enhancing light extraction efficiency and directionality of the emitted light. Although antenna theory assumes coherent emitters, we demonstrate here that the directionality and outcoupling enhancement is also seen for the case of incoherent emitters as in the case of µLEDs. Furthermore, we demonstrate that the outcoupling and collimating performance of the horn antenna is improved by either introducing a discrete or continuously varying descending refractive index layer inside of the Horn. In the presence of such a layer, light from the µLED, which would typically have momentum beyond the escape cone from semiconductor to air, can enter the horn collimator antenna structure. Within this GRIN layer, the pathway of such light is bent, causing a major portion of it to interact with the sidewalls, and is subsequently redirected into a more advantageous direction. In essence, adding the material complexity within the horn leads to the possibility of achieving strong directionality and large outcoupling efficiency enhancement with a smaller geometrical footprint, which is extremely desirable for high-resolution display applications, along with perfect pixel singularization and consequently contrast. In describing the governing mechanisms that lead to outcoupling and directionality enhancement, we first show proof-of-principle 2D calculations. We then proceed to illustrate these principles through 3D cylindrical models, demonstrating the core findings from our 2D calculations. These models are compared against two pertinent reference scenarios: 1) a bare µLED pixel, and 2) a µLED pixel paired with a large, optimized SiO2 half-ellipse lens, where the lens’s diameter is 40 times that of the µLED pixel’s active region and 24 times the pixel’s opening width.

The results show an improvement in $\hbox {LEE}_\text {15}$ of up to one order of magnitude compared to the bare µLED pixel and more than double compared to the µLED pixel with the large SiO2 half-ellipsoidal lens. Achieving such an improvement in total LEE and $\hbox {LEE}_\Gamma$ with minimal geometrical size penalties for real devices would drastically change the prospects of applications of µLEDs in AR/VR and optical communication. To summarize, our key contributions are:

1. The concept of the novel GRIN and µHorn µLEDs and how they improve light extraction.
2. Parameter study for the µHorn µLEDs with different filling materials in 2D.
3. Validation of the µHorn µLED designs in 3D and further investigation into the underlying mechanism which leads to the improvement of efficiency and directionality of 10x compared to the baseline.
4. Discussion and potential outlook for future improvement and applications.

## Theory

**Fig. 1:**
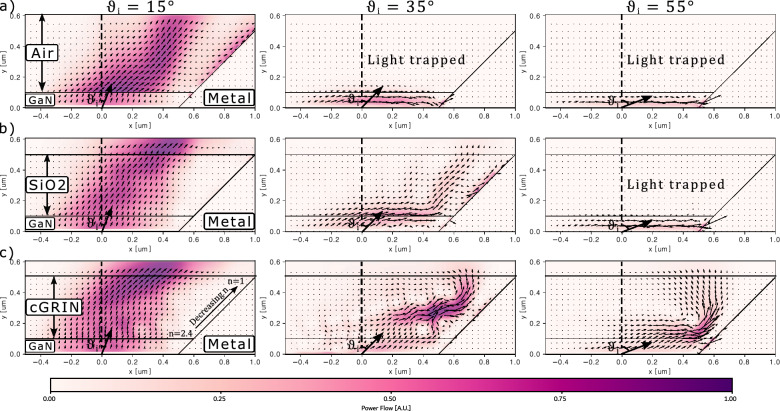
Interaction of Gaussian Beams at different incident angles with the µHorn and different filling materials. 2D model of a portion of the µLED structure under investigation. The colour and length of the arrows indicate the power flow i.e. the real part of the source- and spectrally-weighted, time-averaged poynting vector in arbitrary units of a Gaussian beam launched from the semiconductor side at different angles as it propagates through the µHorn collimator with different filling materials. Note that the model is symmetric around the y-axis but only a part of the geometry is displayed. A Gaussian beam with $\lambda =$ 450nm and injection angles [$15^\circ$, $35^\circ$, $55^\circ$] is injected in different µHorn filling media. Even without filling (row (a)), appropriate sidewalls increase the directionality of the light by redirecting light at the steepest emission angles with respect to the forward direction. Adding an additional filling of SiO2 (row (b)) improves outcoupling but still shows total internal reflection for more oblique angles. By adding a continuous step-down GRIN-style structure (row (c)) inside the µHorn antenna, much less of the light experiences total internal reflection and even more of the emitted light interacts with the sidewalls. Hence the LEE is increased much more than what would be possible via an anti-reflection coating. For the steepest injection angles, it becomes visible that even light which experiences total internal reflection is able to escape the device. In particular, the height h and sidewall angle $\vartheta$ of the µHorn collimator influence how much of the injected power escapes the device and at which angle. An alternative version of this figure with normalized arrow length is shown in subsection A.2..

As outlined in the introduction, this work aims to investigate and enhance the light extraction efficiency (LEE) and directionality of a µLED, specifically focusing on the combined factor of $\eta _\text {LEE} \cdot \eta _\Gamma$.

Fundamentally, an aperture antenna^55^ can be modelled as a Huygens source, which helps in understanding the farfield in terms of diffraction. It is well known that the diffraction of plane waves at a slit aperture results in an intensity distribution given by

1 $$\begin{aligned} I(\theta ; \phi _i) = \text {sinc}\left( \frac{d \pi }{\lambda }(\sin {\theta } \pm \sin {\phi _i})\right) \end{aligned}$$

in the farfield, where $\phi _i$ is the incident angle on the aperture and $\lambda$ the wavelength of the light. Increasing the aperture diameter *d* leads to a more directional farfield. Conversely, a broad incident near-field angular distribution causes a widening of the farfield due to the superposition effect caused by the incident angle. For better illustration, we demonstrate in subsection A.1 how a wide near field angular distribution affects the directionality in the farfield.

Therefore, the emission characteristics of a µLED are inherently constrained by the diffraction of light at the outcoupling region, which acts as an aperture of limited size. More importantly, the light reaching the aperture typically has a broad angular distribution. The broad angular distribution is a consequence of the emission from the quantum wells, which couple to many modes within the µLED. This effect is particularly pronounced in solid-state µLEDs, where the semiconductor layer thickness and lateral size are several times the effective wavelength. Furthermore, these modes are broadened due to the lossy nature of the µLED cavity, which experiences both absorption, which is undesirable, and radiative loss, which is desirable in certain solid angles.

**Fig. 2:**
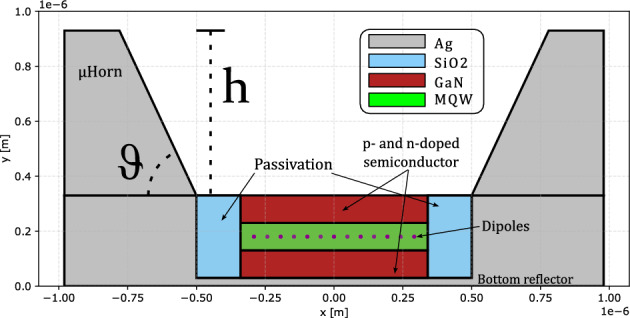
Schematic cross-section of the µLED model investigated in this work. The µHorn height h and the sidewall angle $\vartheta$ are systematically adjusted to determine the combination with the best $\hbox {LEE}_{{15}}$ for different µHorn filling materials. The violet dots represent the position of the quantum well which is approximated by using a fixed amount of dipoles. The substrate is formed by silver which acts as a side and bottom reflector and the MQW together with the doped semiconductor is encapsulated via an electrical passivation of SiO2. We assume that the MQW emission can be represented with a single emission plane at the center of the MQW which would be a valid for a homogeneous recombination probability in the MQWs and the total MQW effective thickness is small compared to the effective wavelength. We treat the MQW with an effective refractive index to represent the optical effect of the quantum well on the emission. The µHorn collimator optical response is insensitive to position of the emitter as the collimation property does not rely on the emission being around a focal point as for the case of lenses or being within near field distance to a particular structure as for photonic crystal-based approaches. For simplicity, we omit the top-side contacting scheme while the bottom contact can be realized via the silver substrate. Further details are given in subsect. 2.2. Note that we consider the y-axis as the emission direction..

Although aperture size is typically application dependent, the angular distribution width can be controlled to some extent through the design of the µLED geometry. For example, managing interference effects with the rear mirror and/or incorporating slanted sidewalls in the µLED can influence this distribution. However, optimizing the sidewalls of a µLED for light extraction and directionality is often challenging. This is because slanted sidewalls, which cannot be properly passivated, may lead to a reduction in internal quantum efficiency. In this work, we propose to further increase the directionality of a µLED by utilizing a µHorn collimator structure on top of the µLED, as shown in Fig. 2 which avoids the necessity to modify the µLED sidewalls.

It is crucial to consider that both the directionality of light and $\eta _\text {LEE}$ play a significant role. However, reducing the angular distribution with a steeper µHorn could potentially impair LEE, necessitating a trade-off or a taller but less steep structure. In the following sections, we will explore how the different geometrical parameters which describe our µHorn collimator: height, opening angle and material fillings, impacts the figure of merit of interest $\eta _\text {LEE}$ and $\eta _\Gamma$.

### GRIN and µHorn interaction with a Gaussian beam

In the simple ray optics picture, a µHorn collimator works by redirecting oblique light propagating within the structure into a small angular cone around the normal direction through interaction with reflective slanted sidewalls. Assuming angularly isotropic emission entering the µHorn collimator, a large geometry is typically needed to ensure that a significant portion of the power traveling in undesirable directions interacts with the slanted sidewalls and is redirected into the desired angular cone. Naturally, not all stray light will be redirected in the desired direction, especially if the target angular cone is small. To some extent, if the µHorn collimator has a smoothly varying surface, the quality of collimation can be improved. However, in general, a large cone is required to significantly increase power in the desired direction.

When the µHorn collimator is unfilled, containing only air, and especially when the µLED pixel is not significantly smaller than the effective wavelength, a considerable amount of light that falls outside the escape cone from the semiconductor to air remains trapped within the µLED (see Fig. 1 (a)) where we show, by utilizing 2D FDTD calculations via the software Lumerical^56^, the power flow, i.e. the real part of the source- and spectrally-weighted, time-averaged Poynting vector of a Gaussian beam at 450 nm wavelength launched from the semiconductor side (GaN) to the interface of the micro-µHorn structure at angles of $15^\circ$, $35^\circ$, and $55^\circ$ from the normal). Since less light enters the µHorn collimator, a larger opening angle and height are generally required to concentrate the limited light that can enter into the desired direction.

When the µHorn is filled with a homogeneous dielectric material, more light from the µLED can enter the µHorn collimator because the escape cone from the semiconductor into the µHorn expands (see Fig. 1 (b)). However, light within the semiconductor-to-air escape cone travels through the dielectric at less oblique angles, reducing interactions with the µHorn’s sidewalls and thus decreasing directionality. Conversely, light with momentum near the edge of the semiconductor-to-dielectric escape cone bends more sharply, interacting more with the µHorn’s sidewalls. This interaction redirects the light favorably, compensating for some loss of directionality. Typically, the MQW in the µLED emits more light at heavily oblique angles outside the semiconductor-to-air escape cone. By filling the µHorn collimator with a dielectric material, light from these angles can be redirected into the desired cone, although the potential for enhancement is limited because redirection is less effective for other momentum regions.

By introducing a gradient-index layer, either discrete (dGRIN) or continuous (cGRIN), we not only allow more light to enter the µHorn collimator but also enable light with varying momenta to follow curved paths at different depths within the µHorn. This interaction with the sidewalls results in more light being redirected into the desired cone. This effect is particularly evident in Fig. 1 c), especially in the middle and right panels, where light outside the primary semiconductor-to-air escape cone, entering at various angles, is significantly bent at different depths and then redirected by the sidewall. Essentially, the power flow is bent within the gradient index until it starts propagating parallel to the interfaces. Just before the radiation starts being redirected downwards, it is intercepted by the side mirror and redirected upwards, rendering it useful for the application and thus increasing the directionality.

In theory, a continuous gradient index material ranging from the maximum refractive index of the µLED down to the refractive index of the ambient medium should achieve the best results for light extraction and redirection. However, in the next sections we demonstrate that even with rough, discrete steps in refractive index, using naturally available materials, a significant enhancement in light outcoupling and directionality can be achieved.

**Fig. 3:**
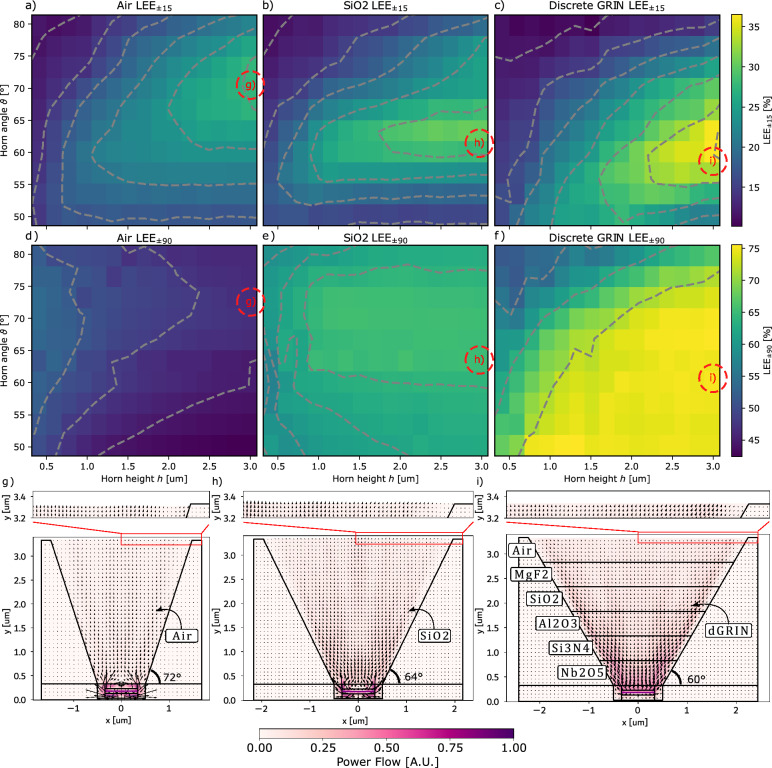
Power flow of the best micro Horn designs for different filler materials and the sweep results of micro Horn angle $\mathbf {\vartheta }$ and micro Horn height h of $\hbox {LEE}_{{15}}$. In (**a**), (**b**), and (**c**), the $\hbox {LEE}_{{15}}$ results are shown over the varying micro Horn height and opening angle for different micro Horn filling materials, while d), (**e**), and (**f**) show the results for $\hbox {LEE}_{{90}}$. (**a**) and (**d**) display the changing LEEs for a filling with air, (**b**) and e) for SiO2, and (**c**) and (**f**) for the discrete GRIN. The best combinations of micro Horn height h and micro Horn angle $\vartheta$ are indicated, and the respective power flow indicated by the length of the arrow in [A.U.] are shown in (**g**), (**h**), and (**i**). Note that the asymmetric appearance of the power flow is only an artifact of plotting the vector fields from left to right. Evidently, the optimal micro Horn height shifts with respect to the height of the device, while the absolute directionality also increases with greater height of the micro Horn in all three cases. However, the optimal angle depends on the filler material. For the air-filled micro Horn, the best result is achieved with $\vartheta =72^\circ$ shown in (**g**), for SiO2 at $\vartheta =64^\circ$ shown in (**h**), and for the discrete GRIN with $\vartheta =60^\circ$ shown in (**i**). Comparing the $\hbox {LEE}_{{15}}$ with the $\hbox {LEE}_{{90}}$ results in (**d**), (**e**), and (**f**), it becomes evident that the filler material has a large impact on the total outcoupling efficiency, which contributes to the forward emission as well. A version of (**g**), (**h**) and (**i**) with emphasized arrow length for the zoomed power flows at the aperture is shown in subsection A.5.

The aforementioned effects occur only when the gradient refractive index layer is thick enough such that light does not simply perceive the entire layer as having a single effective index. The exact thickness at which all the desired effects occur can only be determined through rigorous calculations. Hence, in the following sections, we investigate the effect of the refractive index of various GRIN coatings and other filling materials with respect to the µHorn size and angle.

### Effect of the µHorn collimator on µLED emission

In order to demonstrate how the GRIN-filled horn collimator alters µLED emission characteristics compared to traditional setups, we show how the light within the $\pm 15^\circ$ cone is affected by variations in horn height and angle across three different dielectric filling scenarios via 2D FDTD simulations. For the first case is the µHorn is filled with air, for the second SiO2, and for the third one a GRIN filling which has a discretely varying index from that of GaN to air. Hence, we investigate a simplified µLED structure as shown in Fig. 2 with different materials inside the µHorn. The emission from the quantum well is approximated with dipoles emitting at different positions along the central plane of the active region. For simplicity, the dipole polarization is assumed to be isotropic and we omit the top contact in all cases.

The next step in subsubsection 2.2.4 presents 3D calculations for cylindrical structures at selected parameters to demonstrate that the enhancements observed in these proof-of-principle calculations are also visible in 3D and even more so due to the fact that more light are emitted in oblique directions beyond the escape cone by point dipole sources as compared to line dipole sources.

#### 2D model

The toy µLED model consists of a simplified semiconductor epitaxial stack for the µLED, comprised of a homogeneous layer of GaN. As seen in Fig. 2, the semiconductor thickness is 300 nm and has a width of 600 nm. We also consider that the µLED is placed on top of a silver substrate which surrounds the µLED and acts as a bottom and side reflector. On the sides of the semiconductor, a SiO2 layer is placed, serving as electrical passivation with a width of 200 nm, which is necessary in real chips to electrically separate the p and n doped semiconductor, avoid defect formation and general protection of the active area. The semiconductor region and the sidewall passivation layer thus effectively make a total width of 1 µm, which we consider as the main pixel width where light can escape from (ignoring the metal sidewall thicknesses). Finally, on top of the base structure, the µHorn collimator is placed. For simplicity, the horn collimator reflector is also considered to be comprised of silver.

To gain the most insights into the behaviour of the µHorn, we investigate three different but realistic scenarios:

- In the first scenario, the µHorn is filled with air.
- In the second scenario, it is filled with GaN to match the refractive index of the quantum wells.
- In the third scenario, we implement a discrete GRIN, which is equivalent to a multilayer stack of descending index by using a six-layer step-down coating composed of Nb2O5, Si3N4, Al2O3, SiO2, MgF2, and air, starting with Nb2O5.

For the study, we sweep the µHorn angle from $\vartheta$ [deg] $= [50,\,80]$ and the height h [µm] $= [0.4,\, 3.0]$.

#### ${\textbf {LEE}}_\Gamma$ formulas

To compute the $\hbox {LEE}_{{90}}$ and $\hbox {LEE}_\Gamma$ similarly to Ryu et. al.^57^ we first compute the total emitted farfield by the µLED pixel $\rho _\text {LEE}$ in 2D

2 $$\begin{aligned} \rho _\text {LEE}(\theta , \lambda ) = \frac{n\, c\, \varepsilon _0}{2 P_0(\lambda ) M} \sum _{i=1}^M w_{i, \lambda , \text {pol}} \left| {\textbf{E}}_i(\theta , \lambda ) \right| ^2 \end{aligned}$$

and 3D

$$\begin{aligned} \rho _\text {LEE}(\theta , \varphi , \lambda ) = \frac{n\, c\, \varepsilon _0}{2 P_0(\lambda ) M} \sum _{i=1}^M w_{i, \lambda , \text {pol}} \left| {\textbf{E}}_i(\theta , \varphi , \lambda ) \right| ^2 \end{aligned}$$

where $P_0(\lambda )$ is the energy injected into the simulation, *M* is the number of considered dipoles and $w_{i, \lambda , \text {pol}}$ a weighting factor, *n* the refractive index of the ambient medium, *c* the speed of light and $\varepsilon _0$ the vacuum permittivity. This weighting factor accounts for a dipole position dependent weighting, the spectrum of the emission, and a correction for the contribution to different polarizations. For 2D simulations, the position dependence of dipole emitters does not play a role because the translational symmetry introduces a uniform weighting over the active area. Emission from the edge of the active region sees the same effective emissive area than emission from the center. For 3D calculations, the position dependent weighting accounts for the larger effective emitting area for dipoles towards the outer edge of the quantum well due to the assumption of cylindrical symmetry, see subsect. A.4 for more information. Furthermore, we assume an isotropic power distribution for the different polarizations (i.e. $w_{pol}=[0.33, 0.33, 0.33]$). Note that in this article, the primary emission direction is the y-axis. The spectrum of the emission is assumed to be gaussian and ranges from $\Lambda$ [nm] = [425, 475] with a FWHM of 25 nm to demonstrate that the concept is robust for a typical emission range of a µLED. $E_i(\theta , \varphi , \lambda )$ denotes the electric field in the farfield region for every individual dipole *i* at elevation $\theta$ and azimuth $\varphi$ in spherical coordinates and at wavelength $\lambda$. $\hbox {LEE}_\Gamma$ can be easily computed from $\rho _{\text {LEE}}$ in 2D

3 $$\begin{aligned} {\text {LEE}}_\Gamma = \int _0^\Gamma \textrm{d}\theta \int _{\Lambda } \textrm{d}\lambda \,\rho _{\text {LEE}}(\theta , \lambda ) \end{aligned}$$

and 3D

$$\begin{aligned} \text {LEE}_\Gamma = \int _0^\Gamma \sin {\theta }\, \textrm{d}\theta \int _0^{2\pi }\textrm{d}\varphi \int _\Lambda \textrm{d}\lambda \, \rho _{\text {LEE}}(\theta , \phi , \lambda ). \end{aligned}$$

**Fig. 4:**
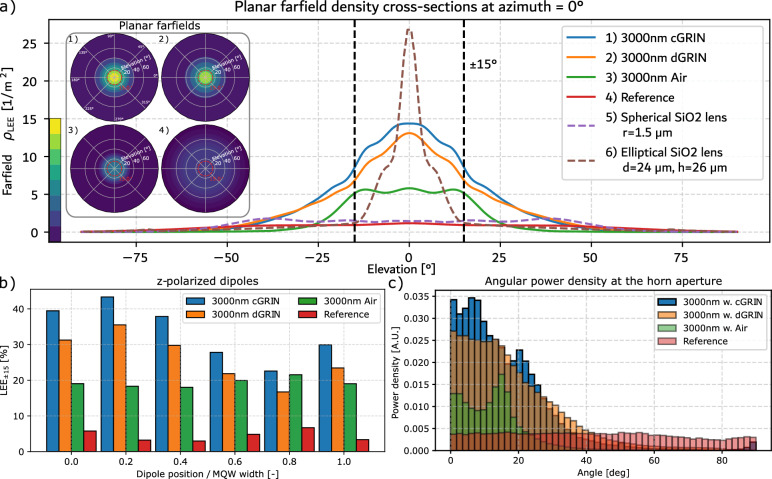
Results and investigation of the 3D cylindrical µLEDs with µHorn and GRIN/air filling compared with the reference pixel and the reference pixel with a small spherical and a tall elliptical lens. The µHorn significantly improves the directionality of the µLED also in 3D. The farfield plots **a)** show the LEE density in the farfield on a plane and, for better visualization, the cross section at azimuth = $0^\circ$. The numerical LEE values are listed in Table 1. Compared to the reference farfield a.4) of the reference pixel (i.e. the pixel without µHorn and any materials, see subsect. A.6), using the µHorn leads to a much brighter and more directional farfield. Furthermore, we added a comparison with a µLED and a small spherical and a a large half-ellipsoidal lens with a diameter of 24µm and a height of 26µm, which provides increased outcoupling due to the lens material having a higher index and focusing the emission through its surface curvature. While a small lens improved directionality only slightly, the large half-ellipsoidal lens can improve directionality significantly but at the cost of a much bigger structure as demonstrated in subsect. A.6. The enhancement via the GRIN becomes evident both in the farfield density plots **a)** as well as **c)** where the distribution of poynting vectors at the opening of the µHorn is shown. Figure c) demonstrates that the distribution of k-vectors in the near field i.e. the magnitude and direction of the optical power (right above the µHorn aperture), is much more confined compared to the reference, however this can only serve as an indication of the directionality without taking the phase of the field into account. In **b)**, the dipole $\hbox {LEE}_\text {15}$ over position in the quantum well for all four cases and z-polarized dipoles is shown, which demonstrates the advantage of the GRIN coating, in particular for the theoretical cGRIN. The remaining polarizations are shown in the supporting information. The $\hbox {LEE}_\text {15}$ remains high for the dipoles furthest from the center of the quantum well which have the highest impact on the total $\hbox {LEE}_\text {15}$ while substantially increasing the $\hbox {LEE}_\text {15}$ in the center of the MQW.

#### 2D proof of principle calculation results

In Fig. 3 (a-c) and (d-f), we illustrate how $\hbox {LEE}_{{15}}$ and $\hbox {LEE}_{{90}}$ vary with the height of the µHorn collimator and its sidewall angle across three distinct filling scenarios. As expected, the highest $\hbox {LEE}_{{15}}$ values are observed with the tallest µHorn collimator, where more light interacts with the sidewall and gets redirected, as evidenced in Fig. 3 (a-c). However, the trends for $\hbox {LEE}_{{90}}$ differ. In the air-filled case, the peak $\hbox {LEE}_{{90}}$ occurs at a lower µHorn height, as shown in Fig. 3 (d). This is because taller structures lead to increased absorption losses at the sidewall, since the filling material has the same refractive index as the ambient environment.

The trend shifts in scenarios involving dielectric fillings. In Fig. 3 (e) and (f), taller µHorns correlate with higher $\hbox {LEE}_{{90}}$ values. This is due to the redirection of light beyond the escape cone of the µHorn’s filling material into the air, significantly boosting both $\hbox {LEE}_{{90}}$ and $\hbox {LEE}_{{15}}$. This behaviour aligns with the Gaussian beam model depicted in Fig. 1 (b) and (c). Notably, in Fig. 3 (e) and (f), the peak $\hbox {LEE}_{{15}}$ values shift to shallower µHorn angles for filled µHorn collimators because more light at large oblique angles must be deflected into a narrower cone within the filling material to match the 15-degree cone in air.

For the third scenario involving a discrete GRIN (Gradient Index) µHorn filling, as shown in Fig. 3 (c) and (f), each layer step must deflect light into a specific angular range to match the 15-degree cone in air. The varying incoming light angles at the sidewalls suggest that each layer might require a unique slope. This complex case merits further investigation, but here we concentrate on elucidating the fundamental principles.

Although the results indicate that a µHorn taller than 3µm would achieve better results, we intentionally omitted even taller structures for practical reasons as that would typically lead to a larger geometrical foot print which is not desirable for high resolution displays and AR/VR applications.

In this considered 2D case, compared to the reference without an outcoupling structure ($\hbox {LEE}_{{15}}$=14.9%, see subsection A.3), the µHorn with a discrete GRIN achieves $\hbox {LEE}_{{15}}$=37.7%, an improvement of 2.5 times. The SiO2-filled µHorn achieves an $\hbox {LEE}_{{15}}$=30.4%, while the air-filled µHorn achieves an $\hbox {LEE}_{{15}}$=27.5%.

To further understand the internal dynamics, we provide a comparison of the power flow, i.e., the time-averaged Poynting vector, incoherently averaged over the dipole positions and weighted by the µLED spectrum, across three scenarios at their highest $\hbox {LEE}_{{15}}$ values (see Fig. 3 (g-i). A notable similarity across the different cases is the power flow distribution along the side wall of the µHorn collimator, with a visible peak at the edge in all scenarios but most pronounced in the GRIN case. This shows the continuous redirection of the light at the sidewalls due to the continuous refraction during propagation. In contrast, the power flow in the center of the outcoupling area is parallel to the normal direction.

For the $\hbox {LEE}_{{90}}$, shown in Fig. 3 (d-f), we observe that $\hbox {LEE}_{{90}}$ is less sensitive to µHorn height and angle. This is because much of the light, traditionally trapped inside the active area due to TIR at the pixel and µHorn collimator interface, is refracted on the metal side walls and redirected out of the structure. The higher outcoupling generally leads to more light in the forward direction, partially explaining the higher $\hbox {LEE}_{{15}}$ for the discrete GRIN stack.

#### 3D cylindrical models

**Table 1:** Comparison of the $\hbox {LEE}_\Gamma$ into the $\pm 15^\circ$ and $\pm 90^\circ$ solid angles for the 3D validation of the 2D parameter sweep results and the improvement ratio to the reference. Its clear that the µHorn significantly improves the directionality. While the µHorn leads to a decrease in the $\hbox {LEE}_{{90}}$ for the air filled case, adding a filling material within the µHorn has a large effect, both on the directional outcoupling as well as total outcoupling. In particular, filling the µHorn with a GRIN coating improves the directionality significantly. For comparison, a small spherical (r=1.5 µm) and a large elliptical (r=12 µm, h=26 µm) SiO2 lens was added on top of the bare reference chip. In particular the large lens leads to a good enhancement of directionality but at the cost of a much larger structure, shown in next to the cGRIN in subsect. A.6. The small spherical lens with a radius of 1.5 µm increases directionality to a small degree but increases the $\hbox {LEE}_{{90}}$. More details about the small lens are presented in subsect. A.6.

Structure	$\hbox {LEE}_{\text {15}}$ $\rightarrow$ vs. Ref.	$\hbox {LEE}_\text {90}$ $\rightarrow$ vs. Ref.	$\hbox {LEE}_\text {15}$ / $\hbox {LEE}_\text {90}$
Reference	2.3% $\rightarrow$ 1.00x	31.8% $\rightarrow$ 1.00x	7%
µHorn w. Air	11.6% $\rightarrow$ 5.04x	27.7% $\rightarrow$ 0.87x	42%
µHorn w. SiO2	11.7% $\rightarrow$ 5.09x	54.1 % $\rightarrow$ 1.70x	22%
µHorn w. GaN	9.2% $\rightarrow$ 4.00x	66.5% $\rightarrow$ 2.09x	13%
µHorn w. dGRIN	19.9% $\rightarrow$ 8.65x	75.6% $\rightarrow$ 2.38x	26%
µHorn w. cGRIN	24.6% $\rightarrow$ 10.57x	80.0% $\rightarrow$ 2.52x	31%
small SiO2 lens	3.0% $\rightarrow$ 1.43x	41.7% $\rightarrow$ 1.31x	8%
large SiO2 lens	11.3% $\rightarrow$ 4.91x	32.6% $\rightarrow$ 1.03x	35%

Having shown the proof-of-principle calculations in 2D, we proceed to show how the principle naturally works in 3D for selected cases assuming cylindrical symmetry. Hence, the results are validated with a 3D simulation of the µLED structure for the best µHorn antenna cases with $\vartheta =60^\circ$ for the GRIN and $\vartheta =72^\circ$ for the air filled case at $h=3$µm to cross check for validity in a realistic scenario. The downside of using 3D simulations is the massive increase of computational power requirements due to the scaling of FDTD with the number of mesh points *n* which is $O(n^4)$^58^.

As the considered structures have cylindrical symmetry, we only need to consider dipoles emitted along a radial cross-section, each with a proper weighting factor contribution to account for the area it represents. The weighting factor ($w_\text {i}$) is essentially described by the expression:

4 $$\begin{aligned} w_\text {i} = \frac{(r_i+\Delta r/2)^2 - (r_i-\Delta r/2)^2}{R^2} \end{aligned}$$

where $r_i$ is the dipole position, $\Delta r$ the distance to the next dipole and *R* the radius of the active area. A graphic explanation of the weighting is given in subsect. A.4. This position-dependent weighting indicates that, in particular, the dipoles towards the outer edges contribute relatively more to the total directional emission and light extraction efficiency, as they represent a larger area coverage of the active area. Most efforts should be directed to enhance the $\hbox {LEE}_\Gamma$ from dipoles located at the sides.

#### 3D results

The results of the cylindrical 3D realizations are presented in Fig. 4 and compared against three reference cases: first, a bare pixel, again with 600nm diameter of GaN region, a 200nm SiO2 sidewall passivation followed by silver sidewalls (The total pixel width opening diameter comprising of GaN+SiO2 passivation is thus again 1um, see subsect. A.6). Second, considering a small, spherical SiO2 lens with $r=1.5$ µm that is similar to the lenses on µLens arrays. Third, considering a large SiO2-optimized half-ellipsoidal lens that is 24 times larger in diameter than the pixel’s width/opening diameter, placed on top of the pixel (see subsect. A.6).

The elliptical lens reference case is assessed using a hybrid approach combining wave and ray optics. Initially, we use rigorous FDTD simulations via the software Lumerical^56^ to compute the far-field emission of a µLED pixel within an SiO2 ambient medium. More details regarding the simulation setup can be found in subsect. A.10. Subsequently, we propagate this farfield as rays through a ray optics simulation tool (LightTools, see subsect. A.6), capturing the portion of light that propagates into the ambient air within the desired cone. In the ray optics simulation, we model the farfield as emanating from a circular surface with a 1 µm diameter, assuming a uniform luminance across the area. This surface is positioned at the bottom center of a half-ellipsoid SiO2 lens. The pixel and lens are further assumed to be placed on top of a silver substrate for simplicity. The 3D simulations corroborate the 2D results, demonstrating significantly higher directionality and $\hbox {LEE}_\text {15}$ compared to the reference case. This outcome is expected, as more light is naturally sent to oblique directions in the 3D case due to solid angle considerations, where there are simply more states/modes that can propagate at oblique polar angles.

Consequently, the impact of the µHorn collimator is more pronounced in 3D than in 2D. We note, however, that the optimal geometrical parameters for the 3D structure can be significantly different from those seen in the 2D case, purely due to the fact that line dipole emission is vastly different compared to point dipole emission. Thus, by no means is the considered 3D structure here optimal. Regardless, these considered 3D realizations already exhibit a very large enhancement of the $\hbox {LEE}_{15}=24.6\%$ of up to 10$\times$ for the cGRIN structure compared to the bare pixel reference and more than twice that of the large SiO2 half-ellipsoidal lens case with $\hbox {LEE}_{15}=11.3\%$ as shown in Fig. 4 a), and 8$\times$ for the smaller spherical lens with $\hbox {LEE}_{15}=11.3\%$. The numerical results are shown in Table 1, which demonstrates that adding an appropriate µHorn increases the directionality for all cases significantly.

**Fig. 5:**
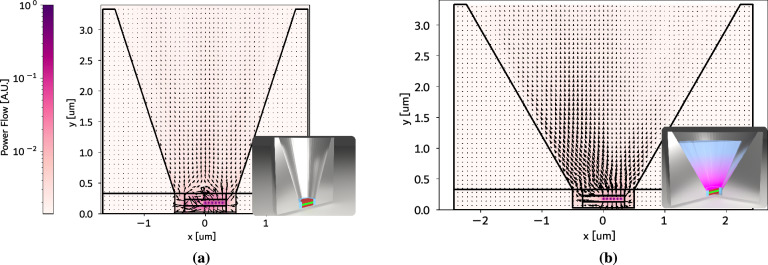
Qualitative comparison of asymmetric power flow in µHorns filled with air and cGRIN. In the 3D power flow depictions, the power has been scaled logarithmically to make the flow more visible since the magnitude of the power is orders of magnitude larger in the vicinity of the dipole emitters than inside the µHorn, especially for the 3D power flows. (**A**) Asymmetric power flow through th eµHorn filled with air.We show the incoherent, weighted superposition of only half ofthe active region. Due to the high refractive index contrast,mostof the power is trapped within the µLED core resulting in the lowLEE90. The steeper angle of the µHorn compared to the cGRINshown in Fig. 5b) leads to a more narrow near field distributionwhich leads to a higher fraction of LEE15/LEE90.However, theabsolute LEE15 is still well be low the LEE15 for thecGRIN, asshown in Table 1. (**B**) Asymmetric power flow through the cGRIN µHorn. We show the incoherent, weighted superposition of only half of the active region. Due to the index match between the cGRIN and the active region, most of the power can escape the µLED core. The power is subsequently reflected at the sidewall on the opposite site of the emission region. After the interception of the emission at the sidewall, a large part of the radiation is redirected and exits the µLED opening close to perpendicular.

**Fig. 6:**
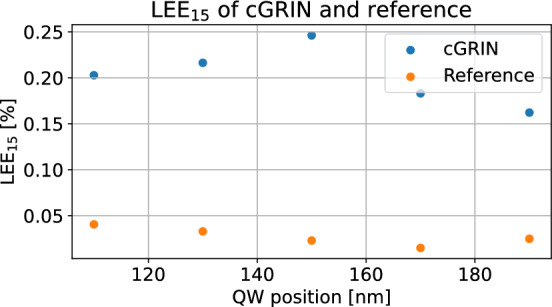
Absolute  $\hbox {LEE}_{{15}}$ of the cGRIN and reference model for different QW positions. The variation is performed from a reference position at 150nm QW position with $\pm 40$ nm. Throughout the position variation, the cGRIN maintains a clear advantage in outcoupling efficiency over the reference chip. This demonstrates that the improvements given by the cGRIN are robust towards changes to the QW position.

In addition to the 3D realization of the dGRIN, we examined the performance of a continuous GRIN (cGRIN) with the same geometric parameters as the dGRIN shown in Fig. 4a) (1) and (2). As anticipated, both directionality and $\hbox {LEE}_{{90}}$ are further enhanced due to the seamless refractive index transition between the semiconductor core of the µLED and the ambient medium. A comparison of the absolute values is provided in Table 1. The reference µLED exhibits low directionality but a comparable $\hbox {LEE}_{{90}}$ to the µHorn filled with air.

We caution the reader that far-field cross sections can be misleading due to the $\sin {\theta }$ factor in Equation 3. This is evident when examining the spherical far-field plots shown in Fig. 4a) or the $\sin {\theta }$ corrected far-field cross-sections shown in subsect. A.7.

Furthermore, a more detailed investigation into the individual dipole efficiencies reveals the reason for the increased absolute efficiency of the GRIN. By resolving the far-field angle-dependent efficiency of the individual dipoles, shown in Fig. 4b), we can observe multiple contributions. For all cases with the µHorn, the $\hbox {LEE}_{{15}}$ of all individual dipoles is increased, hence the much more directional emission and overall higher $\hbox {LEE}_{{15}}$. Interestingly, the dipole $\hbox {LEE}_{{15}}$ of the reference and air-filled µHorn remain relatively stable over the quantum well, while the d- and cGRIN experience a decrease in $\hbox {LEE}_{{15}}$. This is particularly important because, due to the weighting factor of the dipole position, the dipoles towards the outer edge contribute more to the absolute $\hbox {LEE}_\Gamma$. However, the individual $\hbox {LEE}_{{15}}$ remains higher for all dipoles by adding a GRIN filling, which results in an absolute improvement of the $\hbox {LEE}_{{15}}$ for the µLED. Here, we show the efficiency for z-dipoles only. The $\hbox {LEE}_{{15}}$ efficiencies for x- and y-polarization are shown in subsect. A.8 and for $\hbox {LEE}_{{90}}$ in subsect. A.8.

While the analysis of the individual dipole LEE indicates that the light extraction efficiency throughout the quantum well was improved, we can also see the impact of the µHorn collimator in Fig. 4c). As discussed in subsect. 2.2, the presence of the µHorn reduces the width of the angular distribution significantly compared to the almost uniform distribution from the bare reference pixel. Compared to all cases, the cGRIN structure focuses the largest proportion of light into the $\pm 15^\circ$ solid angle. It is also visible that the air-filled µHorn reduces the width of the distribution even further but does not achieve the same amount of total power as the GRIN versions. This is a consequence of the lower overall extraction efficiency and demonstrates the trade-off between increasing directionality at the expense of light extraction efficiency.

The effect of lower extraction efficiency is further exemplified when comparing the asymmetric power flows of the µHorn filled with air in Fig. 5) with those of the µHorn with the cGRIN shown in Fig. 5).

The significant refractive index difference between the active area and the air inside the µHorn results in much lower outcoupling efficiency, which effectively traps most of the light at oblique angles inside the active region. Instead of interacting with the µHorn, this light experiences significant absorption, leading to the lower overall extraction efficiency that occurs in the absence of the GRIN filling within the horn collimator.

To demonstrate the robustness of the enhancement provided by the cGRIN filled µHorn, we show the $\hbox {LEE}_{{15}}$ as a function of the QW position, which is varied by $\pm 40$ nm relative to the centre position we have been considering. The results for both the µLED with cGRIN filled µHorn and the bare µLED reference are shown in Fig. 6. The $\hbox {LEE}_{{15}}$ absolute values and relative enhancement provided by the cGRIN filled µHorn remain large throughout the whole considered 80 nm thickness region and would likely still provide such performance beyond that range of variations. This further supports the conclusion that the cGRIN filled µHorn’s performance does not rely on a position-sensitive resonant effect but instead provides a robust enhancement of $\hbox {LEE}_{{15}}$ over a large thickness range. In subsection subsection A.9, we provide the results directly in terms of relative enhancement $\hbox {LEE}_{15}^\text {cGRIN}$ / $\hbox {LEE}_{15}^\text {reference}$, which shows an enhancement of $\ge 5 \times$ for all considered emitting QW positions.

## Conclusion

We have shown the impact of a Horn collimator with different material fillings on the emission characteristics of a simplified µLED model. The proposed GRIN-filled Horn significantly enhances both the total light extraction efficiency within the $\pm 15^\circ$ cone by an order of magnitude compared to the bare µLED pixel and more than doubles it compared to the case of a µLED pixel with a vastly large half-ellipsoidal SiO2 lens, whose diameter is 40 times the active area, as confirmed by full 3D FDTD simulations. Depending on the choice of material that fills the Horn, the optimal opening angle is shifted, which is a consequence of the trade-off between maximizing the directionality and the total extraction efficiency. Regardless, the enhancement is achieved with a comparatively small geometrical footprint which could lead to monochromatic dpi’s of up to 6500ppi, making it ideal for many display and augmented reality (AR) applications^4,5^ or optical communications^6,7^.

This improvement can be attributed to a combination of factors: the enhancement of outcoupling efficiency over the active region, driven by the refractive index / impedance matching of the GRIN with the active region, and the improved redirection of emission at the Horn collimator. This results in a narrow angular distribution in the near field and subsequent a directional farfield which would enhance coupling of the light source to the secondary optics/waveguides for AR/VR. As a consequence of better coupling and higher efficiency, improving $\hbox {LEE}_{{15}}$ would lead to lower power consumption and subsequently lower self-heating^20^ for the same display brightness, reducing some of the most critical limitations for µLED displays^59^.

### Outlook

The investigation presented in this article identifies an approach to achieving ultra-high LEE and directional µLEDs, although it is not exhaustive. Significant improvements and geometrical optimizations are still possible, which could further enhance directionality and outcoupling efficiency^60–64^. Specifically, geometrical designs that maintain high coupling efficiency while producing a narrower angular distribution could result in even higher $\hbox {LEE}_\Gamma$ than demonstrated in this work and due to the small geometric size, additional secondary collimation techniques such as µlens arrays could be added to increase the efficiency even further.

Possible geometrical optimizations of the core µHorn include the following areas of investigation:

- We assumes a particularly simple µLED core geometry but the core and the interaction of the dipoles with the surrounding has a large effect on the outcoupling efficiency and directionality^30^. Altering the core therefore could enhance the $\hbox {LEE}_\Gamma$.
- We assumed a linear decrease in refractive index for the GRIN, which is straightforward. However, it remains unclear whether a different refractive index distribution might be beneficial, such as a quadratically decreasing profile, which is often used in optical gradient index fibres.
- The µHorn angle was assumed to be constant throughout this article although the results of this work already show that the optimal µHorn angle depend on the filling material/refractive index. Investigating a variable angle Horn profile might yield significant improvements over the current linear profile.

Experimental realization of the proposed concept is the focus of ongoing work. In practice, one must likely approximate the ideal continuous-GRIN profile using a stack of discrete layers composed of readily available materials; as demonstrated above, even these stepped-index implementations deliver substantial enhancement. One fabrication route would be to build the multilayer horn directly on the µLED, by alternating deposition and selective etch-back of the different dielectric materials to form the GRIN sidewall. Alternatively, one could first deposit the dielectric stack onto a planar µLED wafer, then perform a back-side etch through to the top surface to define both the pixel and the horn sidewall, finishing with a conformal metal deposition that serves as both electrical contact and reflective cladding. Determining the optimal process flow and material choices for high-volume manufacturing remains an important topic for future research.

For red µLEDs based on InGaAlP-where the semiconductor refractive index is inherently high, and a smooth, continuous or discrete index gradient may be difficult to realize due to the lack of suitable materials-one promising strategy is to combine the micro-Horn geometry with sub-wavelength surface texturing. Such hybrid approaches could approximate a graded refractive index while leveraging well?established lithographic texturing techniques, opening the door to efficient, highly directional emission across the full RGB spectrum.

## Data Availability

The data supporting the findings of this study are available from the corresponding author upon reasonable request. Due to competitive concerns, specific models and explicit details have not been disclosed. However, we are committed to transparency and will provide the necessary data to qualified researchers who seek to verify our results or conduct further analysis. Please contact the corresponding author to discuss access to the data.
